# Hypoglycemic Properties of *Leccinum scabrum* Extracts—An In Vitro Study on α-Glucosidase and α-Amylase Inhibition and Metabolic Profile Determination

**DOI:** 10.3390/jof10100718

**Published:** 2024-10-15

**Authors:** Valeria Ferraro, Anna Spagnoletta, Natalie Paola Rotondo, René Massimiliano Marsano, Daniela Valeria Miniero, Gaetano Balenzano, Annalisa De Palma, Alessandro Colletti, Maria Letizia Gargano, Giovanni Lentini, Maria Maddalena Cavalluzzi

**Affiliations:** 1 Department of Pharmacy-Pharmaceutical Sciences, University of Bari Aldo Moro, 70125 Bari, Italy; valeria.ferraro@uniba.it (V.F.); natalie.rotondo@uniba.it (N.P.R.); giovanni.lentini@uniba.it (G.L.); mariamaddalena.cavalluzzi@uniba.it (M.M.C.); 2 Laboratory “Regenerative Circular Bioeconomy”, ENEA-Trisaia Research Centre, 75026 Rotondella, Italy; anna.spagnoletta@enea.it; 3 Department of Biosciences, Biotechnology, and Environment, University of Bari Aldo Moro, 70125 Bari, Italy; renemassimiliano.marsano@uniba.it (R.M.M.); danielavaleria.miniero@uniba.it (D.V.M.); annalisa.depalma@uniba.it (A.D.P.); 4 Department of Medicine & Surgery, LUM University Giuseppe Degennaro Torre Rossi, Piano 5 S.S. 100 Km. 18, 70010 Casamassima, Italy; 5 Department of Soil, Plant and Food Sciences, University of Bari Aldo Moro, 70125 Bari, Italy; gaetano.balenzano@uniba.it; 6 Department of Drug Science and Technology, University of Turin, 10124 Torino, Italy

**Keywords:** *Leccinum scabrum*, α-glucosidase, α-amylase, medicinal mushrooms, mycochemicals, fatty acids, GC-MS, HRMS, Boletaceae, type-2 diabetes

## Abstract

Type-2 diabetes affects an increasing percentage of the world’s population and its control through dietary management, involving the consumption of health-promoting foods or their derived supplements, is a common strategy. Several mushroom species have been demonstrated to be endowed with antidiabetic properties, resulting from their ability in improving insulin sensitivity and production, or inhibiting the carbohydrate-hydrolyzing enzymes α-amylase and α-glucosidase. This study aimed to investigate for the first time the hypoglycemic properties of the edible mushroom *Leccinum scabrum* (Bull.) Gray. Mushroom extracts were prepared through the microwave-assisted extraction (MAE) technique using green solvents with different polarity degrees. The inhibition activity of all the obtained extracts on both α-glucosidase and α-amylase was evaluated and the highest activity was observed for the EtOAc extract which showed an IC_50_ value about 60-fold lower than the reference compound 1-deoxynojirimycin (DNJ) on α-glucosidase (0.42 ± 0.02 and 25.4 ± 0.6 µg/mL, respectively). As expected on the basis of the literature data concerning both α-glucosidase and α-amylase inhibition, a milder inhibition activity on pancreatic α-amylase was observed. Preliminary in vivo tests on *Drosophila melanogaster* carried out on the most active obtained extract (EtOAc) confirmed the in vitro observed hypoglycemic activity. Finally, the EtOAc extract metabolic profile was determined through GC-MS and HRMS analyses.

## 1. Introduction

Edible mushrooms are widely consumed in diets all over the world due to their intense flavor and taste, as well as their high nutritional value, which qualifies them as functional foods [1,2,3,4,5]. The growing importance also lies in their content of a wide spectrum of bioactive metabolites responsible for multiple medicinal and health-promoting properties, making mushrooms an ideal ingredient in mycotherapy products, nutraceuticals, food supplements, and fortified foods and beverages [3,6,7,8,9]. Polysaccharides, especially β-glucans, terpenoids and triterpenoids, fatty acids, polyphenols, sterols, proteins, peptides, and lectins have been reported to be present in the fruiting bodies of edible and medicinal mushrooms, resulting in several beneficial effects on human and animal health [9,10,11,12,13]. It has been widely demonstrated that both wild-growing and cultivated mushroom species are endowed with important pharmacological properties, such as antioxidant, anti-inflammatory, neuroprotective, hypocholesterolemic, antimicrobial, and anticancer, to mention a few [10,14,15,16,17,18]. Furthermore, the antidiabetic properties of different fungal species, especially within the genus *Ganoderma* P. Karst., one of the most studied, as well as in many others such as *Pleurotus* (Fr.) P. Kumm., *Agaricus* L., *Grifola* Gray, and *Lentinula* Earle, were also described [19,20,21,22,23,24,25,26,27]. In vitro and in vivo studies demonstrated that their extracts, either as a crude extract or as isolated bioactive compounds, are effective in controlling blood glucose levels as well as boosting insulin production or sensitivity [9,25,28,29,30].

Type 2 diabetes is a severe metabolic disorder currently considered to be among the highest health risk factors worldwide. The resulting increase in postprandial blood glucose and a persistent condition of hyperglycemia can lead to serious long-term complications such as hypertension, diabetic nephropathy, neuropathy, retinopathy, and cardiovascular diseases [9,31,32]. The drugs currently available, such as acarbose and miglitol, are very effective in controlling glycemic levels as they can inhibit the intestinal and pancreatic enzymes responsible for the hydrolysis of the carbohydrates taken in with diet, namely α-glucosidase and α-amylase. Unfortunately, gastrointestinal side effects often limit their use in routine clinical practice [33].

We aimed to evaluate for the first time the antidiabetic properties of *Leccinum sca*-*brum* (Bull.) Gray, whose antimicrobial activity has already been established [34]. This basidiomycete, belonging to the family Boletaceae Chevall., is a mycorrhizal species mainly widespread in the Northern Hemisphere where it prefers deciduous forests, especially in association with birch, hence the name ‘birch bolete’, but also occurs under beech trees [34,35,36]. *L*. *scabrum* ranks among the wild-growing mushrooms most widely appreciated as food in Scandinavia, as well as Central and Eastern Europe, due to its organoleptic properties and notable nutritional value [34,37]. To date, only a few investigations have dealt with this mushroom species, recently pointing out its content in health-promoting compounds as well as antimicrobial, antioxidant, and cytotoxic properties on cancer cells [34,35,37,38,39,40]. Considering the alarming spreading of metabolic syndrome, a survey was undertaken to assess the possible hypoglycemic effect of *L*. *scabrum* to possibly identify novel mushroom-based dietary supplements or functional foods useful to maintain healthy glycemic levels and prevent the onset of type 2 diabetes, even avoiding the uncomfortable contraindications associated with current oral hypoglycemic agents.

## 2. Materials and Methods

### 2.1. Chemicals and Reagents

Solvents used for extraction, all reagents and buffers for enzymatic tests, α-glucosidase (EC 3.2.1.20) from *Saccharomyces cerevisiae* (recombinant, lyophilized powder, ≥100 units/mg protein), porcine pancreatic α-amylase (EC 3.2.1.1; Type VI-B, ≥5 units/mg solid), 4-nitrophenyl-α-d-glucopyranoside (*p*-NPG), starch azure, 1-deoxynojirimycin (DNJ), acarbose, and Geduran^®^ Si 60 for column chromatography (0.063–0.200 mm) were purchased from Sigma-Aldrich (Milan, Italy).

Dulbecco’s modified Eagle’s medium high glucose (DMEM), fetal bovine serum (FBS), penicillin, l-glutamine, trypsin and streptomycin were purchased from Euroclone (Italy); 3-(4,5-dimethylthiazol-2-yl)-2,5-diphenyltetrazolium bromide (MTT) was provided from Invitrogen (Thermo Fisher Scientific, Waltham, MA, USA). Human hepatoma HepG2 cells were purchased from the American Type Culture Collection (ATCC), Rockville, MD, USA.

### 2.2. Fungal Material and Extract Preparation

Powder of dried basidiomes of *Leccinum scabrum* (Bull.) Gray proceeding from the Białowieża Forest (BLR, POL) was provided by the Herbarium of the Department of Agricultural, Food and Forest Sciences, University of Palermo, Italy (SAF 545) [34].

Basidiomes of *Ganoderma lucidum* (Curtis) P. Karst. were obtained from Alphay International Inc. of Nantong (China) and ground into powder using a special mill.

#### 2.2.1. Microwave-Assisted Extraction (MAE)

A CEM Discover Bench Mate microwave reactor equipped with Synergy software (SKU 909100) was employed to carry out a closed-system MAE at a constant temperature, with continuous stirring. The temperature was measured and controlled by a built-in infrared detector. Six solvents with different polarity degrees were used. For each sample, in a microwave tube, 70 mg of *L*. *scabrum* powder was suspended in 2.1 mL of the appropriate solvent [solid/solvent ratio 1:30 (*w*/*v*)] and then irradiated with microwaves (30–100 W according to the used solvent) at 80 °C for 5 min. After filtration, the solution was centrifuged at 8000 rpm for 10 min and the supernatant recovered. Finally, the solvent was evaporated to dryness under reduced pressure on a rotary evaporator to obtain a solid, which was stored at –20 °C until needed for analysis.

As regards *G*. *lucidum*, extracts were prepared as described above, using chloroform and ethyl acetate as extraction solvents.

#### 2.2.2. Conventional Extraction

A suspension of 490 mg of *L*. *scabrum* powder in 14.7 mL of EtOAc [solid/solvent ratio 1:30 (*w*/*v*)] was stirred at room temperature (25 °C) for 24 h. The solution was then filtered and processed as above described.

### 2.3. α-Glucosidase Inhibitory Activity Assay

The spectrophotometric method described by Tavani et al. (2017) was used to determine the inhibition of α-glucosidase activity of the fungal extracts [41]. Briefly, the enzyme was dissolved in 445 μL of 10 mM phosphate buffer (PBS 1X, pH 7.28) at a final concentration of 2.5 U/mL and incubated at 37 °C for 5 min along with 5 μL of sample in DMSO at the appropriate concentration. The reaction was triggered by adding 50 μL of the substrate 4-nitrophenyl-α-d-glucopyranoside (*p*-NPG, final concentration 80 μM) and stopped after 10 min with 500 μL of 0.5 M Na_2_CO_3_. The amount of 4-nitrophenol released from *p*-NPG was quantified as absorbance at 400 nm. Variations in absorbance values were obtained by comparison with a control, in which 5 μL of DMSO replaced the sample solution. DNJ, a known α-glucosidase inhibitor, was used as a reference standard. The percentage inhibition of enzyme activity was calculated by means of the following formula:

$$Inhibitory\;activity\;\left(\%\right)=\frac{A_{C}-A_{S}}{A_{C}}\times 100$$

where *A_C_* and *A_S_* stand for absorbance of control (100% enzyme activity) and sample test, respectively.

The α-glucosidase inhibitory activity of extracts was expressed as the inhibitory concentration required for 50% inhibition of the enzyme activity (IC_50_). Eight gradual extract concentrations below and above the IC_50_ values, approximately estimated in previous experiments, were tested. In each set of experiments, the assay was performed in triplicate and repeated three times.

### 2.4. α-Amylase Inhibitory Activity Assay

The α-amylase inhibitory activity was performed as described by Tamil et al. (2010) with modifications [42]. The substrate was prepared by suspending 10 mg/mL (1% *w*/*v*) of starch azure in 0.5 M Tris–HCl buffer (pH 6.9) containing 0.01 M CaCl_2_ and boiling for 5 min with continuous stirring. The suspension was then dispensed in 0.2 mL aliquots into eppendorf tubes (1.5 mL) and incubated in a water bath at 37 °C for 5 min. Thereafter, 0.2 mL of 0.5 M Tris–HCl buffer containing 5 μL of extract in DMSO at the appropriate concentration was added. The reaction was started by adding 0.1 mL of porcine pancreatic α-amylase in 0.5 M Tris–HCl buffer (2 U/mL) to the tube containing the substrate solution and mushroom extract. After incubating at 37 °C for 10 min, the reaction was stopped by the addition of 0.5 mL of 50% acetic acid. Tubes were then centrifuged at 8000 rpm for 5 min at 4 °C and the absorbance of the solution was measured at 595 nm. Samples were compared for absorbance values with a positive control, in which 5 μL of DMSO replaced the sample solution. Acarbose, a well-known α-amylase inhibitor, was used as a reference standard. The percentage of enzyme inhibition was calculated by applying the following formula:

$$Inhibitory\;activity\;\left(\%\right)=\frac{\left(A_{C+}-A_{C-}\right)-\left(A_{S}-A_{B}\right)}{\left(A_{C+}-A_{C-}\right)}\times 100$$

where *A_C_*_+_, *A_C_*_−_, *A_S_*, and *A_B_* indicate the absorbance of 100% enzyme activity (positive control), 0% enzyme activity (negative control without enzyme), sample test (with enzyme), and sample blank (a test sample without enzyme), respectively. Each assay was performed in triplicate and repeated three times.

### 2.5. In Vitro Cytotoxicity Assay

The cell viability assay was carried out using a human hepatoma cell line (HepG2) and following the protocol described in Cavalluzzi et al. (2022) [43]. HepG2 cells were cultured in Dulbecco’s modified Eagle’s medium (DMEM) high glucose supplemented with 10% (*v*/*v*) heat-inactivated fetal bovine serum (FBS), 2 mM L-glutamine, 100 U/mL penicillin (100 U/mL) and 100 μg/mL streptomycin at 37 °C in an atmosphere of 5% CO_2_. After growth, cells were detached with trypsin 0.2%, centrifuged at 1500 rpm for 10 min, plated in a 96-well plate at the concentration of 4.5 × 10^4^ cell/well, and incubated at 37 °C, 5% CO_2_ for 24 h. Hence, having reached 70–80% confluence, the cells were incubated for 24 h in the presence of *L*. *scabrum* extracts at a concentration from 1 to 100 μg/mL. Cell viability was evaluated by the MTT test, by measuring the formazan proportionally produced. After medium removal, an MTT solution in PBS 1X (0.5 mg/mL) was added to each well for 2 h. Hence, the medium was then substituted by DMSO to dissolve the formazan product and absorbance values were measured at 545 nm with a multilabel plate counter Victor3 V (Perkin Elmer, Milan, Italy). Triplicate cultures were set up for each tested concentration.

### 2.6. In Vivo Assessment of Hypoglycemic Activity of L. scabrum Microwave/EtOAc Extract

The Bacup W80 wild-type *Drosophila melanogaster* strain used in this study was maintained under standard conditions on cornmeal medium in a constant climate chamber with a temperature of 18 °C, 60% relative humidity, and a 12 h day/night cycle.

For experimental testing, 3–6-day-old males were anesthetized and starved for 7 h before being exposed to the food source. Liquid food was presented using microcapillaries (1.1 mm diameter) loaded with a 50 g/L sucrose solution, with or without the drugs. The meniscus of the solution within the capillary was marked with the purpose of determining food consumption after treatment. In total, 20–200 µL pipette tips were cut at the end to fit and hold the glass capillaries. Six pipette tips holding the capillaries were positioned into a polyurethane plug that fit into the opening of a plastic vial (8 cm height, 3.3 cm diameter). Forty individuals per vial were tested.

Drugs were diluted in the 50 g/L sucrose solution as follows:

- Acarbose and DNJ working concentrations: 50 µM, 100 µM, 150 µM, and 200 µM;
- Mushroom microwave/EtOAc extract stock solution (10 mg/mL) was diluted to 0.3%, 0.1%, 0.05%, 0.025%, and 0.0125%.

After 24 h of exposure, dead individuals (if any) were recorded and removed from the pool. Glucose quantification was performed according to Tennessen et al. (2014) [44], with some modifications. Flies were rinsed several times with 1 mL cold PBS to remove all traces of food from the exterior, then homogenized in 160 µL PBS using a glass Teflon Dounce homogenizer. The homogenized tissues were centrifuged for 2 min at 13000 rpm at 4 °C, and 10 µL of the supernatant was applied to the reactive strip of a OneTouch Verio glucometer to measure glucose levels.

### 2.7. Statistical Method

In both enzyme inhibitory activity and cytotoxicity determination, the assay was performed in triplicate and repeated three times. The average values were recorded, and all results were expressed as mean ± standard deviation (SD).

Data were processed through the GraFit 7 data analysis software to construct the corresponding dose-response curves and thus enable IC_50_ values to be obtained.

### 2.8. Qualitative Extract Characterization—Fractionation, GC-MS Analysis, and HRMS-ESI Analysis of L. scabrum Extract Obtained with EtOAc under Microwave Irradiation

#### 2.8.1. Fractionation and GC-MS Analysis

EtOAc crude extract (65.6 mg) was purified by column chromatography on silica gel by eluting with EtOAc/hexane 2:8. The eluting solution was collected in successive aliquots of 500 μL each. By monitoring the progress of fractionation by thin layer chromatography (TLC silica gel 60 F254, Merck KGaA, Darmstadt, Germany) and checking spots both at UV light (254 nm) and after treatment with iodine, aliquots were merged and three fractions were obtained: LS1 (17.09 mg), LS2 (10.32 mg), and LS3 (18.28 mg), the latter fraction having been eluted in gradient from EtOAc/hexane 2:8 to EtOAc.

Secondary metabolites of LS1–LS3 fractions were then separated using an HP–5MS capillary column (30 m × 0.25 mm, i.d. 0.25 μm) (Agilent, Santa Clara, CA, USA). In total, 1 μL sample was injected using a split ratio of 1:100. The column was held at 70 °C for 3 min after injection, the temperature programmed at 3 °C/min to 280 °C, and held for 20 min more. Helium was used as carrier gas, at a constant column flow rate of 1.5 mL/min. The injector temperature was 250 °C, and the detector temperature was 230 °C. The mass spectrometer was operated at 70 eV with a mass range from 30 to 400 atomic mass units (AMU). Secondary metabolites were identified by comparing their retention times and mass spectra with those available in the National Institute of Standards and Technology (NIST) Mass Spectral Library Version NIST 11 and/or reported in data from the NIST standard reference database (NIST chemistry webbook) and in the literature. Only compounds with a NIST similarity of at least 80% were considered. Results were expressed as the individual relative percentage of each secondary metabolite present in the sample.

#### 2.8.2. HRMS-ESI Analysis

HRMS analyses were performed using a microTOF QII mass spectrometer (Bruker Daltonics, Bremen, Germany) equipped with ESI operating in both positive and negative ion modes. The methanol-soluble fraction of the extract was analyzed. Mycochemicals present in mushrooms of various species and reported from the literature and the MeFSAT database (Medicinal Fungi Secondary Metabolites and Therapeutics) were searched for. Mass spectra were acquired both in the positive and negative ion modes in the mass range of 100–650 *m*/*z* and compared with literature data to carry out a tentative identification of bioactive compounds. Where no suitable spectrometry literature references were available, the metabolites that were supposed to be possibly present in the extract were tentatively determined through mass spectral fragmentation evaluation.

## 3. Results

### 3.1. Biological Assays

Six solvents with increasing lipophilicity were used to prepare *L*. *scabrum* extracts, initially working under microwave irradiation (80 °C, 5 min). Their α-glucosidase inhibitory effect was then evaluated and a potency increase was registered passing from polar to apolar solvents (Table 1), with the only exception of 2-methyl tetrahydrofuran (2-Me THF) extract whose IC_50_ value (0.99 ± 0.23 μg/mL) was more than 2-fold higher than that obtained with ethyl acetate (EtOAc) extract (0.42 ± 0.02 μg/mL). An IC_50_ value twice as high as that of the microwave-treated extract was obtained in the case of maceration (room temperature, 24 h).

Our experiments also confirmed the ability of *G*. *lucidum* to inhibit α-glucosidase (Table 1), as previously described in the literature [45]. In particular, the MAE technique and two different solvents, namely chloroform and ethyl acetate, were used and the same inhibition activity was observed, having obtained 5.1 ± 0.3 μg/mL and 4.9 ± 1.6 μg/mL as IC_50_ values, respectively. In both cases, the *G*. *lucidum* extract was about one order of magnitude less potent than the *L*. *scabrum* extract obtained under microwave irradiation with EtOAc as the extraction solvent.

Finally, regardless of both the extraction solvent used and the technique adopted, our extracts always showed an α-glucosidase inhibition activity higher than DNJ, used as a reference compound.

With regard to α-amylase, acarbose was chosen as the reference compound, and its IC_50_ value was determined (38 ± 6 μg/mL, Table 1). Results were expressed as the inhibition percentage registered at 100 μg/mL, and a dose-dependence was observed within the investigated range of concentrations. Furthermore, in agreement with what was observed for α-glucosidase, the α-amylase inhibition activity of *L*. *scabrum* extracts increased with the extraction solvent lipophilicity, and the ethyl acetate extract showed the greatest effect. Surprisingly, the extract obtained by maceration with EtOAc showed the same α-amylase inhibition activity (31.0 ± 2.2%) registered for the microwave-irradiated one (28.0 ± 0.1%).

Cytotoxicity was assessed on the HepG2 cell line (Table 1) and all MA extracts were not cytotoxic at the highest tested dose (100 μg/mL), except for the 2-Me THF one which showed an IC50 value of 2.45 ± 0.12 μg/mL. The EtOAc macerate proved to be weakly toxic, its IC50 value being 16.2 ± 1.8 μg/mL.

The hypoglycaemic activity of the most active microwave/EtOAc extract was also in vivo assessed on *Drosophila melanogaster*, according to the literature procedure [44], and acarbose and DNJ were used as references. At the highest tested concentration (200 μM), acarbose was more effective than DNJ (percentage inhibition of 47% and 16%, respectively). The microwave/EtOAc extract, tested in the range of concentrations 0.0125–0.3 mg/mL, showed a clear inhibitory effect on the carbohydrate-hydrolyzing enzymes with the maximum inhibitory activity of about 43% having been observed.

### 3.2. Extraction Efficiency

The highest extraction yields were reached with polar solvents (70% EtOH and 70% iPrOH, Table 2), in agreement with the high content of water-soluble polysaccharides, such as β-glucans, in mushrooms [12]. Furthermore, *G*. *lucidum* showed a lower extraction yield than *L*. *scabrum*, when the same solvent (EtOAc) and technique (MAE) were used.

### 3.3. GC-MS Profiling of L. scabrum Ethyl Acetate Extract Fractions

The *L*. *scabrum* extract obtained under microwave irradiation and using EtOAc as the extraction solvent was endowed with the highest inhibition activity on both α-glucosidase and α-amylase, and was also devoid of cytotoxicity on HepG2 cells at the same concentration; thus, it was evaluated for its chemical composition.

**Figure 1:**
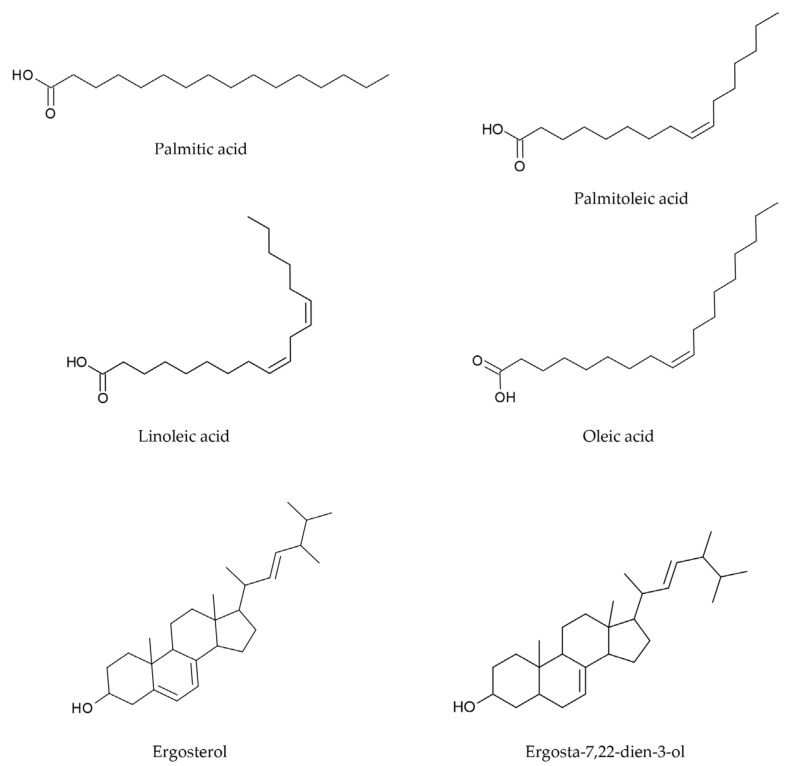
Molecular structure of the major fatty acids and sterols detected by GC-MS in fractions LS1, LS2, and LS3 of the microwave/EtOAc extract of *L*. *scabrum*.

Firstly, the crude extract underwent column chromatography purification which led to the collection of three pooled fractions, namely LS1 (17.09 mg), LS2 (10.32 mg), and LS3 (18.28 mg). Each fraction was then screened for its chemical profile through GC-MS analysis. The metabolite identification was carried out by matching the mass spectral fragmentation patterns of each revealed analyte with those of known compounds in the NIST library or previously described in the literature. The identified metabolites, their retention time, the relative peak area (%), key ion species, and the chemical class they belong to are listed in Table 3. For each analyzed fraction, all the compounds are arranged according to their elution progression. Figure 1 illustrates the molecular structure for the major compounds detected in fractions LS1, LS2, and LS3.

Although the volatile component of the ethyl acetate extract of *L*. *scabrum* is a mixture of 21 compounds belonging to different chemical classes, a clear predominance of fatty acids was observed in all chromatographic fractions, with palmitic, linoleic, and oleic acids having been detected in all three.

Based on the peak area evaluation, approximately 98% of the compounds detected in LS1 were tentatively identified, with 80.16% being fatty acids and small amounts of fatty acid esters (6.78%), acyclic alkenes (8.03%), triterpenes, spiro compounds, and phenol analogues. Fatty acids also prevailed in LS2, making up 81% of the total detected components, followed by 7.65% of fatty acid esters. In addition to a high level of fatty acids (35.96%), an abundance of fatty acid esters (18.99%), acyclic alkenes (17.87%), and sterols (15.52%), namely ergosterol and its analogues, was recorded in the LS3 fraction, with the remaining 6.2% consisting of phenol analogues, fatty alcohols, spiro compounds, and benzoquinones.

### 3.4. HRMS-ESI Analysis of L. scabrum Ethyl Acetate Extract

Mycochemicals contained in the ethyl acetate extract were also tentatively identified through their HRMS spectra. The proposed compounds, along with their chemical class, the ionization mode, theoretical and experimental mass, and key fragments are listed in Table 4. A total of 11 metabolites were tentatively identified.

Several fatty acids were detected with HRMS analysis, in agreement with the GC-MS results, together with three alkaloids, namely 4-[2-Formyl-5-(hydroxymethyl)-1H-pyrrol-1-yl]butanoic acid, pyrrolezanthine, and leccinine A, with the latter being a typical *Leccinum* spp. secondary metabolite. Finally, the sphingolipid 1,2-diacetylsphingosine and the ergostanoid gymnasterone C were also identified.

## 4. Discussion

This study investigated the potential hypoglycemic effect of the edible mushroom *L*. *scabrum* by evaluating the inhibition activity of its extracts against α-glucosidase and α-amylase, the key enzymes involved in the carbohydrate digestion process.

Conventional extraction methods of secondary metabolites from natural sources usually require long extraction times as well as large amounts of organic solvents. To overcome these drawbacks and efficiently extract bioactive compounds from *L*. *scabrum* powder, the green microwave-assisted extraction technique (MAE) was applied. Based on our previous experience [46,47,48], the extraction process was carried out at 80 °C for 5 min. To investigate the effect of the extraction solvent polarity on both extraction yield and biological activity, and to recover the widest range of bioactive compounds to screen, extractions from *L*. *scabrum* were performed using six solvents of increasing lipophilicity, starting from 70% EtOH up to 2-MeTHF. It is worth highlighting that only green solvents were used [49,50]. Based on the obtained results, we can state that the extraction yield increased in direct proportion to the polarity of the solvent used, being maximum with 70% EtOH (37.2%) and minimum with EtOAc (5.0%).

Conversely, significantly stronger inhibition of α-glucosidase activity was observed when apolar solvents were used to carry out the extraction under microwave irradiation, with EtOAc extract giving the lowest IC_50_ value reached within the solvent series (0.42 ± 0.02 μg/mL). The same trend was observed when the extracts were tested against α-amylase. Notably, due to the low α-amylase inhibition activity observed, higher extract concentrations than those used for the α-glucosidase inhibition assay were necessary to possibly establish the α-amylase inhibition IC_50_ values. However, precipitation occurred at concentrations higher than 1000 μg/mL and the IC_50_ values were not determined. Therefore, the results were expressed as the inhibition percentage observed at 100 μg/mL, with this value being the highest dose reached in the extract’s cytotoxicity evaluation. Our results were in agreement with what is reported in the literature, with the α-amylase inhibition generally being observed at concentrations much higher than those required to inhibit α-glucosidase [19,20,51,52]. Furthermore, as above described for α-glucosidase, the extraction solvent lipophilicity was directly related to the α-amylase inhibition activity, with EtOAc still being the solvent giving the most active extract. These findings suggest that the mushroom metabolites mainly responsible for the observed effects are nonpolar compounds.

Having identified EtOAc as the best extraction solvent among those tested under MAE conditions, maceration with the same solvent was carried out to compare the two techniques and evaluate the influence of microwave irradiation on the extraction process. No significant difference in terms of extraction yield was observed, whereas the inhibition activity observed for the EtOAc/microwave extract was two-fold stronger on α-glucosidase, but similar on α-amylase, in comparison with macerate (Table 2). The latter, as well as being less powerful, was obtained with an extremely long procedure lasting approximately 300 times longer than MAE. Moreover, with the same solvent used (EtOAc), only the macerate extract exhibited cytotoxicity on HepG2 cells, even at low concentrations (IC_50_ = 16.2 ± 1.8 μg/mL), thus suggesting that long contact times between solvent and mushroom powder can cause the release or the formation of cytotoxic substances. These findings confirmed that MAE guarantees the production of potent and non-toxic extracts, as well as guaranteeing a dramatic reduction of both extraction times and energy costs, thus being an excellent secondary metabolites extraction technique from natural sources [43].

With the aim of comparing the anti-glucosidase activity of *L*. *scabrum*, never been investigated so far, with a mushroom with similar properties previously described in the literature, *G*. *lucidum* was chosen. In 2011, Fatmawati et al. [45] reported the extract obtained by 24-h maceration in chloroform at 25 °C as capable of reducing enzyme activity, with an IC_50_ value of 109.6 ± 1.4 μg/mL. Surprisingly, our chloroform extract prepared through microwaves showed greater potency (IC_50_ = 5.1 ± 0.3 μg/mL), which was also confirmed when EtOAc was used (IC_50_ = 4.9 ± 1.6 μg/mL), once again shedding light on the MAE procedure. By comparing the IC_50_ values obtained for the two mushroom species extracted with EtOAc, the greater effect of *L*. *scabrum* in comparison with *G. lucidum* is evident. Finally, both *L*. *scabrum* and *G*. *lucidum* extracts always showed an α-glucosidase inhibition activity higher than DNJ, used as a reference compound.

Having detected the most potent inhibition activity for the EtOAc/microwave extract of *L*. *scabrum*, a preliminary in vivo evaluation of its hypoglycemic effect on *D*. *melanogaster* was carried out. In individuals fed with the mushroom extract, a consistent lowering of glucose levels, up to a 43% decrease compared to the control, was observed. Having unequivocally demonstrated the in vivo hypoglycemic effect of *L*. *scabrum*, more in-depth studies will be undertaken.

The EtOAc/microwave extract of *L*. *scabrum* chemical composition was also investigated through chromatographic and spectrometric techniques. The qualitative characterization of the EtOAc/extract through GC-MS and HRMS-ESI revealed the presence in *L*. *scabrum* of a variety of compounds endowed with multiple interesting biological activities. The most abundant among them were fatty acids, especially linoleic, oleic, and palmitic acids. In particular, our results confirm, as observed for other species, the high dominance in *L*. *scabrum* of linoleic acid, an essential polyunsaturated fatty acid, followed by the monounsaturated oleic and saturated palmitic acids. The unsaturated palmitoleic acid and the saturated stearic acid were found in lower amounts. On the other hand, the high content of saturated and unsaturated fatty acids in *Leccinum* spp. and other mushroom genera, such as *Ganoderma*, *Pleurotus*, *Boletus*, *Lentinula*, *Agaricus*, and *Lactarius*, among others, has been previously reported in the literature [34,53,54,55,56,57,58,59]. Conceivably, just fatty acids should greatly contribute to the observed hypoglycemic activity of our extracts. In fact, in addition to a variety of evidenced biological activities, such as anti-inflammatory [60,61,62], antimicrobial and antibiofilm [61,63,64], antioxidant [57,61,63,64], anticancer [61,64,65], hypocholesterolemic, hypolipidemic [66,67], and many others, they also show anti-diabetic properties stemming from the ability to improve insulin synthesis and sensitivity [60,68] and to inhibit α-amylase and even more α-glucosidase activities [19,31,69,70,71,72].

Fatty acid derivatives were also detected in *L*. *scabrum* EtOAc extract (Table 3), specifically fatty acid esters and fatty alcohols, previously reported as metabolites of other mushroom species. Particularly, 9,12-octadecadienoic acid methyl ester, a linoleic acid ester, 9-octadecenoic acid methyl ester, an oleic acid ester, and the more abundant benzenepropanoic acid 3,5-bis(1,1-dimethylethyl)-4-hydroxy octadecyl ester identified in the EtOAc extract of *L*. *scabrum* had already been described in *Pleurotus*, *Ganoderma* and *Trametes* genera of medicinal mushrooms [53,73,74,75]. The fatty alcohol *n*-nonadecan-1-ol, although observed in small quantities, has been previously reported in methanolic extracts of *Pleurotus ostreatus* and the edible bolete *Phlebopus beniensis* [75,76].

The identified 2,4-bis(1,1-dimethylethyl)phenol has also been previously reported in several mushroom species, among which *Trametes versicolor*, *Lentinula edodes*, and *Agaricus bisporus* [77,78].

As regards sterols, both ergosterol and its analogue ergosta-7,22-dien-3-ol accounted for about 14% of LS3 fraction, thus being the third most abundant secondary metabolite in EtOAc extract, just after linoleic acid (28.4%) and benzenepropanoic acid 3,5-bis(1,1-dimethylethyl)-4-hydroxy-, octadecyl ester (19%). As it is well known, ergosterol is the most abundant sterol in the cell membrane of mushrooms, including *L. scabrum* [37,79], and is a crucial component in the process of vitamin D2 (ergocalciferol) synthesis, the latter being useful for human nutrition. In fact, after being transported to the kidney, ergocalciferol is further transformed into 1,25-dihydroxycholecalciferol, also known as calcitriol, which plays a pivotal role in calcium homeostasis and bone health [55,80]. Notably, in vivo studies have already been reported describing the ergosterol anti-diabetic properties [80], thus supporting the possible hypoglycemic effect of *L*. *scabrum* extracts. About 3% of ergosta-7,22-dien-3-ol (stellasterol) was found in the extract, with its anti-glucosidase activity being already described in the literature [81]. Finally, the identified neoergosterol has been described as a component of the sterol fraction of *Cortinarius xiphidipus* [82].

To investigate the composition of the non-volatile component of the EtOAc microwave extract of *L*. *scabrum*, a qualitative characterization was carried out using high-resolution mass spectrometry (HRMS). As regards fatty acids, the HRMS analysis confirmed the GC-MS results. Palmitoleic, palmitic, linoleic, oleic, and stearic acids were detected, together with 9-hydroxy-10,12-octadecadienoic acid, previously described in the literature as detected in *Ganoderma* species [83,84]. Three alkaloids were also identified, namely leccinine A, pyrrolezanthine, and 4-[2-formyl-5-(hydroxymethyl)-1*H*-pyrrol-1-yl]butanoic acid, previously recorded in the genus *Leccinum*, with leccinine being its peculiar metabolite as the name suggests [85,86]. Notably, α-glucosidase inhibition activity of pyrrole alkaloids isolated from the medicinal mushroom *Grifola frondosa* has already been described in the literature, with the strongest activity, even higher than both sterols and acarbose, having been observed for pyrrolezanthine [32]. It is therefore reasonable to assume that pyrrole alkaloids, rather than ergosterols, were responsible for the anti-α-glucosidase effect of *G*. *frondosa* extract and that the same could also be true for *L*. *scabrum*. For the first time, we report on the presence of some secondary metabolites in *L*. *scabrum* which were previously found in diverse mushrooms: 1,2-diacetylsphingosine, described by Choi et al. (2013) for the edible *Grifola gargal*, and the ergostanoid gymnasterone C, counted among the ergosterol derivatives ubiquitous in fungi [87,88].

Notably, based on this metabolic profiling, many other biological activities could be hypothesized for the *L*. *scabrum* mushroom. Antifungal, antibacterial, and antioxidant properties have been reported for 9,12-octadecadienoic acid methyl ester and benzenepropanoic acid 3,5-bis(1,1-dimethylethyl)-4-hydroxy octadecyl ester, the latter being present in large amounts in all three analyzed fractions [53,66,75]. 9-Octadecenoic acid methyl ester, detected in edible mushrooms as *P. ostreatus* and *Lentinus squarrosulus*, exerts antioxidant, anti-inflammatory, antiandrogenic, cancer preventive, and dermatitigenic effects [75,89]. The fatty alcohol *n*-nonadecan-1-ol is known for exhibiting good anti-inflammatory, antimicrobial, and cytotoxic activities [90,91].

2,4-Bis(1,1-dimethylethyl)phenol is endowed with multiple biological activities, such as antibacterial, antifungal, antiviral, anticancer, antioxidant, anti-inflammatory and many others [77,78]. Moreover, the literature reported several health-promoting effects of ergosterol, such as anti-cancer, immunostimulating, anti-inflammatory, neuroprotective, antioxidant, antimicrobial, hypocholesterolemic, and protective from cardiovascular diseases [14,80,82,92,93]. Furthermore, neoergosterol showed significant cytotoxic activity on several tumor cell lines [82].

GC-MS analysis also revealed the presence of significant amounts of alkenes, previously detected in several species of mushrooms and possessing interesting biological activities. Among them, 7-hexadecene and 1-octadecene, possessing antioxidant, antimicrobial, and anticancer activities [53,94], were the most abundant. 9-Eicosene and 5-eicosene, previously reported in *G*. *lucidum* and *S*. *squarrosulus*, were identified, with the former having been described as endowed with antimicrobial, antituberculosis, and cytotoxic properties, and the latter antibacterial effects [53,89].

Leccinine A showed protective activity against endoplasmic reticulum stress-dependent cell death, whereas 4-[2-formyl-5-(hydroxymethyl)-1H-pyrrol-1-yl]butanoic acid can stimulate RAW264.7 macrophage cells increasing the production of NO, TNF-α and IL-12, in addition to a moderate anti-tyrosinase, anti-glucosidase and radical scavenging activity, and hepatoprotective properties [32,95,96,97]. Finally, 9-hydroxy-10,12-octadecadienoic acid showed an antagonistic effect against plant pathogenetic fungi [83,84].

In conclusion, the green microwave-assisted extraction procedure allowed us to obtain an *L*. *scabrum* extract endowed with an interesting inhibition activity of α-amylase and α-glucosidase, the main enzymes involved in the carbohydrate digestion process at the intestinal level, and therefore related to the postprandial increase in blood glucose level. An effective strategy to prevent diabetes mellitus and contrast hyperglycemia is to inhibit the activity of both enzymes or at least α-glucosidase, usually inhibited at lower concentrations than α-amylase, to protract the carbohydrate digestion and therefore retard the glucose absorption. The main bioactive compounds responsible for the observed effect were tentatively identified through GC-MS and HRMS analyses and the results agreed with what was previously reported in the literature for other mushroom species. Therefore, in addition to its high nutritional value and the medicinal properties so far described for this mushroom species, *L*. *scabrum* proved to be promising for the treatment of diabetes type-2, possibly representing a natural adjuvant in oral antidiabetic therapy.

## Figures and Tables

**Table 1:** In vitro α-Glucosidase and α-amylase inhibitory activities and cytotoxicity assessment of extracts from basidiomes of *Leccinum scabrum* and *Ganoderma lucidum*.

Mushroom	Extraction Method	Solvent	α-Glucosidase	α-Amylase		Cytotoxicity
			IC_50_ (μg/mL) ^1,2^	IC_50_ (μg/mL) ^1,2^	Inhibition (%) at 100 μg/mL^2^	IC_50_ (μg/mL) ^1,2^
*L*. *scabrum*	MAE	70% EtOH	13.0 ± 0.4	>1000	11 ± 3	>100
	MAE	70% *i*PrOH	8.8 ± 2.2	>1000	9 ± 2.74	>100
	MAE	EtOH	2.67 ± 0.19	>1000	13.44 ± 0.18	>100
	MAE	*i*PrOH	2.1 ± 0.5	>1000	16.9 ± 1.3	>100
	MAE	EtOAc	0.42 ± 0.02	>1000	27.96 ± 0.08	>100
	MAE	2-MeTHF	0.99 ± 0.23	>1000	25.5 ± 1.6	2.45 ± 0.12
*L*. *scabrum*	Maceration	EtOAc	0.87 ± 0.23	>1000	30.9 ± 2.2	16.2 ± 1.8
*G. lucidum*	MAE	CHCl_3_	5.1 ± 0.3	-	-	-
	MAE	EtOAc	4.9 ± 1.6	-	-	-
DNJ	Reference inhibitor		25.4 ± 0.6 ^a^	-	-	-
Acarbose	Reference inhibitor		-	38 ± 6 ^b^	-	-

^1^ Half-maximal inhibitory concentration. ^2^ Values are presented as mean ± SD (*n* = 3). ^a^ Lit: 203 ± 9 μM; 33.1 ± 1.5 μg/mL [41]. ^b^ Lit: 83.33 ± 0.34 µg/mL [42].

**Table 2:** Extraction yields.

Entry	Mushroom	Extraction Method	Solvent	Weight of Extract Dry Residue (mg) ^a^	Yield (%) ^b^
1	*L. scabrum*	MAE	70% EtOH	26.0	37.2
2	*L. scabrum*	MAE	70% *i*PrOH	23.2	33.1
3	*L. scabrum*	MAE	EtOH	11.9	17.0
4	*L. scabrum*	MAE	*i*PrOH	7.1	10.1
5	*L. scabrum*	MAE	EtOAc	3.5	5.0
6	*L. scabrum*	MAE	2-MeTHF	4.9	6.9
7	*L. scabrum*	maceration	EtOAc	23.7	4.8
8	*G. lucidum*	MAE	CHCl_3_	2.6	3.7
9	*G. lucidum*	MAE	EtOAc	2.3	3.3

^a^ Amount of dry residue obtained starting from 0.070 g of dry mushroom powder in the case of MAE and 0.49 g in the case of maceration. ^b^ Yield% = (weight of the dry extract/weight of the dry mushroom powder) × 100.

**Table 3:** Bioactive compounds identified by GC-MS analysis in fractions LS1, LS2, and LS3 of *Leccinum scabrum* ethyl acetate extract.

Fraction	No.	Compound	Molecular Formula	Mol. Mass (Nominal; g/mol)	RT ^a^ (min)	Peak Area (%)	Key Ion Species *m*/*z*	Chemical Class
LS1	1	2,4-Bis(1,1-dimethylethyl)phenol (2,4-Di-*tert*-butylphenol)	C_14_H_22_O	206	28.815	2.31	191 (100), 206 (M^+^, 15)	Phenol analogues
	2	7-Hexadecene	C_16_H_32_	224	31.102	4.33	55 (100), 224 (M^+^, 2.3)	Acyclic alkene
	3	1-Octadecene	C_18_H_36_	252	36.541	3.70	83 (100), 252 (M^+^, 1.7)	Acyclic alkene
	4	7,9-Di-*tert*-butyl-1-oxaspiro[4.5]deca-6,9-diene-2,8-dione	C_17_H_24_O_3_	276	39.704	0.45	57 (100), 276 (M^+^, 3.9)	Spiro compounds
	5	9-Hexadecenoic acid (Palmitoleic acid)	C_16_H_30_O_2_	254	40.469	1.06	55 (100), 254 (M^+^, 1.6)	Fatty acid (monounsaturated)
	6	*n*-Hexadecanoic acid (Palmitic acid)	C_16_H_32_O_2_	256	41.170	13.42	73 (100), 256 (M^+^, 20)	Fatty acid (saturated)
	7	9,12-Octadecadienoic acid methyl ester	C_19_H_34_O_2_	294	43.812	0.13	67 (100), 294 (M^+^, 4.6)	Fatty acid esters (linoleic acid ester)
	8	9-Octadecenoic acid methyl ester (Methyl oleate)	C_19_H_36_O_2_	296	43.957	0.13	55 (100), 265 (M^+^ −31, 9.3)	Fatty acid esters (oleic acid ester)
	9	9,12-Octadecadienoic acid (Linoleic acid)	C_18_H_32_O_2_	280	45.030	49.37	67 (100), 280 (M^+^, 15.8)	Fatty acid (polyunsaturated, (essential)
	10	9-Octadecenoic acid (Oleic acid)	C_18_H_34_O_2_	282	45.770	14.91	55 (100), 264 (M^+^ −18, 15.8)	Fatty acid (monounsaturated)
	11	Octadecanoic acid (Stearic acid)	C_18_H_36_O_2_	284	46.004	1.40	55 (100), 284 (M^+^, 20.4)	Fatty acid (saturated)
	12	(all-*E*)-2,6,10,15,19,23-Hexamethyl-2,6,10,14,18,22-tetracosahexaene (Squalene)	C_30_H_50_	410	58.164	0.08	69 (100), 410 (M^+^, 4.4)	Triterpene
	13	Benzenepropanoic acid 3,5-bis(1,1-dimethylethyl)-4-hydroxy-,octadecyl ester	C_35_H_62_O_3_	530	75.260	6.53	57 (100), 530 (M^+^, 80.2)	Fatty acid ester
LS2	1	9-Hexadecenoic acid (Palmitoleic acid)	C_16_H_30_O_2_	254	40.400	0.50	55 (100), 236 (M^+^, −18, 4.6)	Fatty acid (monounsaturated)
	2	*n*-Hexadecanoic acid (Palmitic acid)	C_16_H_32_O_2_	256	40.975	10.18	73 (100), 256 (M^+^, 18.4)	Fatty acid (saturated)
	3	9,12-Octadecadienoic acid (Linoleic acid)	C_18_H_32_O_2_	280	44.980	46.16	67 (100), 280 (M^+^, 7.7)	Fatty acid (polyunsaturated, essential)
	4	9-Octadecenoic acid (Oleic acid)	C_18_H_34_O_2_	282	45.180	24.17	55 (100), 264 (M^+^ −18, 3.0)	Fatty acid (monounsaturated, essential)
	5	Benzenepropanoic acid 3,5-bis(1,1-dimethylethyl)-4-hydroxy-,octadecyl ester	C_35_H_62_O_3_	530	75.032	7.65	57 (100), 530 (M^+^, 45)	Fatty acid ester
LS3	1	2,5-Cyclohexadiene-1,4-dione, 2,6-bis(1,1-dimethylethyl)- (2,6-Di-*tert*-butyl-*p*-benzoquinone)	C_14_H_20_O_2_	220	27.373	0.38	177 (100), 220 (M^+^, 50.9)	Benzoquinone
	2	2,4-Bis(1,1-dimethylethyl)phenol (2,4-Di-*tert*-butylphenol)	C_14_H_22_O	206	28.817	4.42	191 (100), 206 (M^+^, 16.3)	Phenol analogues
	3	7-Hexadecene	C_16_H_32_	224	31.104	6.82	55 (100), 224 (M^+^, 1.0)	Acyclic alkene
	4	1-Octadecene	C_18_H_36_	252	36.541	5.84	83 (100), 252 (M^+^, 8.0)	Acyclic alkene
	5	7,9-Di-*tert*-butyl-1-oxaspiro[4.5]deca-6,9-diene-2,8-dione	C_17_H_24_O_3_	276	39.702	0.67	57 (100), 276 (M^+^, 3.0)	Spiro compounds
	6	*n*-Hexadecanoic acid (Palmitic acid)	C_16_H_32_O_2_	256	40.922	2.92	73 (100), 256 (M^+^, 15.7)	Fatty acid (saturated)
	7	9-Eicosene	C_20_H_40_	280	41.476	3.52	57 (100), 280 (M^+^, 1.1)	Acyclic alkene
	8	*n*-Nonadecan-1-ol	C_19_H_40_O	284	43.560	0.72	83 (100), 224 (M^+^ −60, 1.0)	Fatty alcohol
	9	9,12-Octadecadienoic acid (Linoleic acid)	C_18_H_32_O_2_	280	44.970	28.39	67 (100), 280 (M^+^, 8.5)	Fatty acid (polyunsaturated, essential)
	10	9-Octadecenoic acid (Oleic acid)	C_18_H_34_O_2_	282	45.116	4.65	55 (100), 264 (M^+^ −18, 8.5)	Fatty acid (monounsaturated, essential)
	11	5-Eicosene	C_20_H_40_	280	46.001	1.70	55(100), 280 (M^+^, 1.1)	Acyclic alkene
	12	Ergosta-5,7,22-trien-3-ol (Ergosterol)	C_28_H_44_O	396	64.245	11.26	69 (100), 396 (M^+^, 59.4)	Sterols
	13	Ergosta-7,22-dien-3-ol	C_28_H_46_O	398	64.442	2.98	69 (100), 398 (M^+^, 19.3)	Sterols
	14	Neoergosterol	C_27_H_40_O	380	64.701	0.62	237 (100), 380 (M^+^, 34.4)	Sterols
	15	Ergosta-7-en-3-beta-ol	C_28_H_48_O	400	65.508	0.66	43 (100), 400 (M^+^, 63.6)	Sterols
	16	Benzenepropanoic acid 3,5-bis(1,1-dimethylethyl)-4-hydroxy-,octadecyl ester	C_35_H_62_O_3_	530	75.177	18.99	57 (100), 530 (M^+^, 61.1)	Fatty acid ester

^a^ RT = retention time.

**Table 4:** Tentative compounds identified in ethyl acetate extract of *Leccinum scabrum* by HRMS under negative ([M − H]^−^) and positive ([M + H]^+^, [M + Na]^+^) ionization.

Class	Proposed Compound	Molecular Formula	Ionization Mode	Calculated Mass	Experimental Mass	Key Fragments *m*/*z* (% rel. abund.)
Pyrrole alkaloids	4-[2-Formyl-5-(hydroxymethyl)-1*H*-pyrrol-1-yl]butanoic acid	C_10_H_13_NO_4_	[M − H]^−^	210.0771	210.0771	94.0295 (100), 124.0390 (20.42)
	5-Hydroxymethyl-1-[2-(4-hydroxyphenyl)-ethyl]-1*H*-pyrrole-2-carbaldehyde (Pyrrolezanthine)	C_14_H_15_NO_3_	[M + H]^+^	246.1124	246.1047 ^a^	105.0699 (100), 125.0150 (11.61)
Alkaloids	Ethyl [*N*-(2-phenylethyl)formamido]acetate (Leccinine A)	C_13_H_17_NO_3_	[M + Na]^+^	258.1100	258.1307 ^a^	171.0623 (100), 105.0693 (70.31)
Fatty acids	9-Hexadecenoic acid (Palmitoleic acid)	C_16_H_30_O_2_	[M − H]^−^	253.2173	253.2165	237.2200 (100), 196.0977 37.1) 58.0039 (43.2)
	*n*-Hexadecanoic acid (Palmitic acid)	C_16_H_32_O_2_	[M − H]^−^	255.2329	255.2323	255.2327 (100), 58.0276 (15.81)
	9,12-Octadecadienoic acid (Linoleic acid)	C_18_H_32_O_2_	[M − H]^−^	279.2330	279.2326	279.2326 (100) 261.2236 (11.25), 127.0763 (8.29), 96.9702 (37.86)
	9-Octadecenoic acid (Oleic acid)	C_18_H_34_O_2_	[M − H]^−^	281.2486	281.2480	265.22536 (100), 281.2480 (59.4)
	Octadecanoic acid (Stearic acid)	C_18_H_36_O_2_	[M − H]^−^	283.2642	283.2612	283.2609 (100), 155.3271 (11.82)
	9-Hydroxy-10,12-octadecadienoic acid	C_18_H_32_O_3_	[M − H]^−^	295.2278	295.2279	183.0100 (100), 277.2118 (15)
Sphingolipids	1,2-Diacetylsphingosine	C_22_H_43_NO_4_	[M + Na]^+^	408.3084	408.3139 ^a^	322.3064 (100), 88.1102 (23.51)
Ergostanoid	Gymnasterone C	C_28_H_40_O_2_	[M − H]^−^	407.2956	407.2998	255.2323 (100) 151.0602 (20.98)

^a^ Despite a high mass error, spectral fragmentation study confirmed the compound structure, as reported in Supporting Information Figures S1–S3.

## Data Availability

The data presented in this study are available within the article.

## Supplementary Materials

The following supporting information can be downloaded at https://www.mdpi.com/article/10.3390/jof10100718/s1. Figure S1: HRMS spectrum and mass spectral fragmentation study of the metabolite tentatively identified as 5-hydroxymethyl-1-[2-(4-hydroxyphenyl)-ethyl]-1*H*-pyrrole-2-carbaldehyde (pyrrolezanthine); Figure S2: HRMS spectrum and mass spectral fragmentation study of the metabolite tentatively identified as ethyl [*N*-(2-phenylethyl)formamido]acetate (leccinine A); Figure S3: HRMS spectrum and mass spectral fragmentation study of the metabolite tentatively identified as 1,2-diacetylsphingosine.
