# Supplementary material for: Hypoglycemic Properties of Leccinum scabrum Extracts—An In Vitro Study on α-Glucosidase and α-Amylase Inhibition and Metabolic Profile Determination
Source: J Fungi (Basel). 2024 Oct 15;10(10):718. doi: 10.3390/jof10100718 (PMC11508548; doi:10.3390/jof10100718)
Supplement: Supplementary file 1 [file jof-10-00718-s001.zip › jof-3214316-supplementary.pdf]

Supplementary Materials

Hypoglycemic Properties of *Leccinum scabrum* extracts—An In Vitro Study on *α*-Glucosidase and *α*-Amylase Inhibition and Metabolic Profile Determination

Table of contents:

1. **Figure S1.** HRMS spectrum and mass spectral fragmentation study of the metabolite tentatively identified as 5-hydroxymethyl-1-[2-(4-hydroxyphenyl)-ethyl]-1*H*-pyrrole-2-carbaldehyde (pyrrolezanthine).
2. **Figure S2.** HRMS spectrum and mass spectral fragmentation study of the metabolite tentatively identified as ethyl [*N*-(2-phenylethyl)formamido]acetate (leccinine A).
3. **Figure S3.** HRMS spectrum and mass spectral fragmentation study of the metabolite tentatively identified as 1,2-diacetylsphingosine.

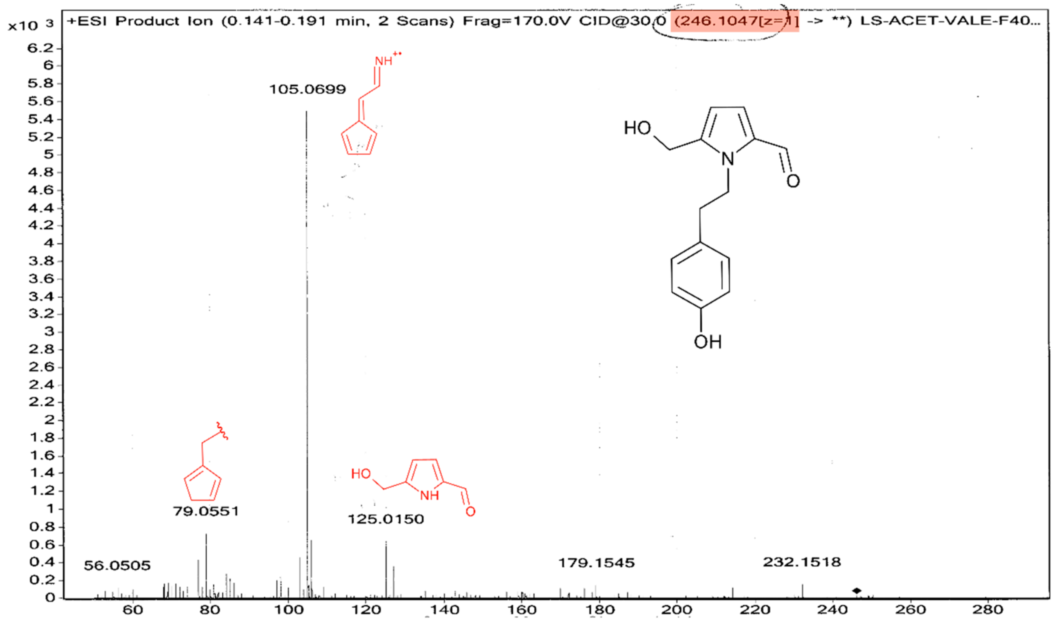

Figure S1. HRMS spectrum and mass spectral fragmentation study of the metabolite tentatively identified as 5-hydroxymethyl-1-[2-(4-hydroxyphenyl)-ethyl]-1*H*-pyrrole-2-carbaldehyde (pyrrolezanthine).

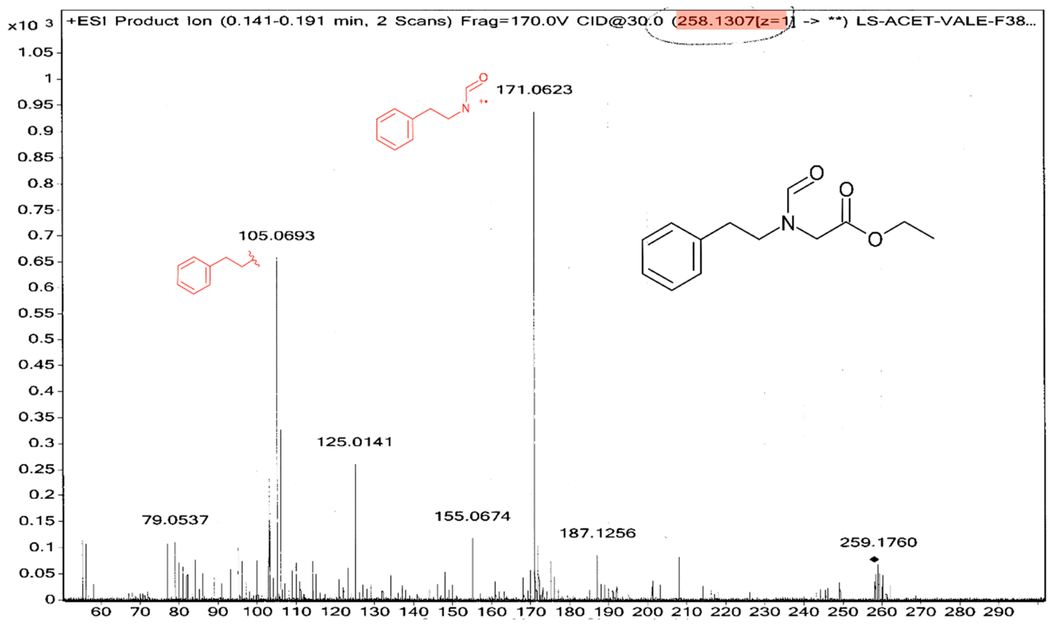

Figure S2. HRMS spectrum and mass spectral fragmentation study of the metabolite tentatively identified as ethyl [*N*-(2-phenylethyl)formamido]acetate (leccinine A).

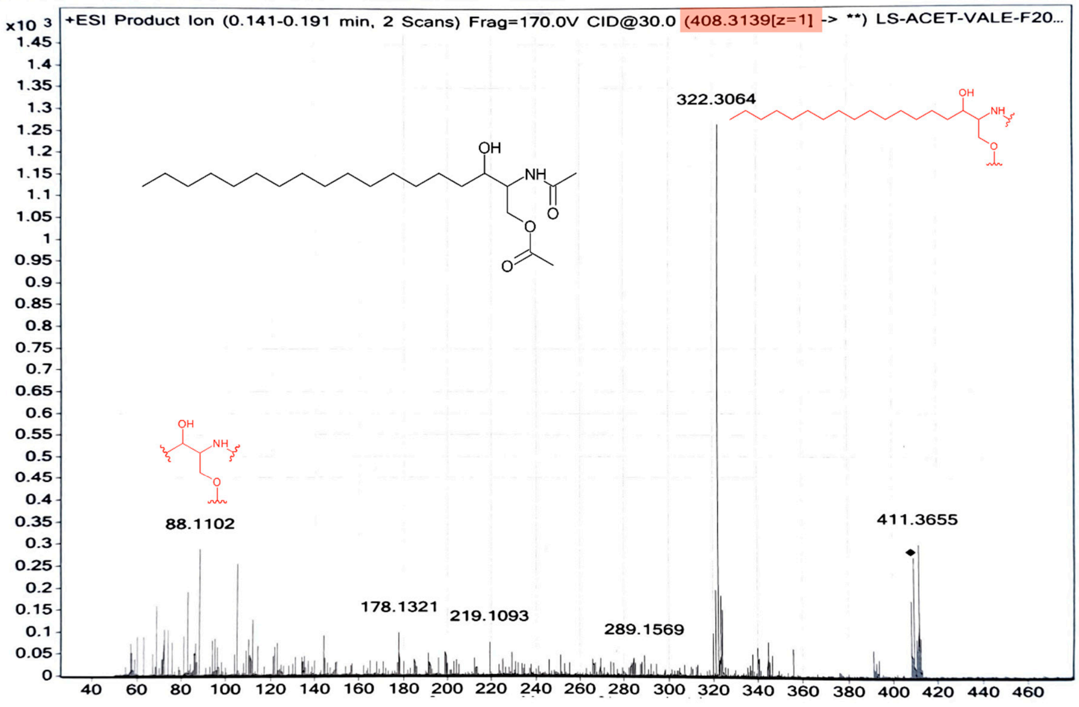

Figure S3. HRMS spectrum and mass spectral fragmentation study of the metabolite tentatively identified as 1,2-diacetylsphingosine.
